# Molecular Mechanisms Governing Sight Loss in Inherited Cone Disorders

**DOI:** 10.3390/genes15060727

**Published:** 2024-06-01

**Authors:** Chloe Brotherton, Roly Megaw

**Affiliations:** 1 MRC Human Genetics Unit, Institute of Genetics and Cancer, University of Edinburgh, Crewe Road, Edinburgh EH4 2XU1, UK; c.c.brotherton@sms.ed.ac.uk; 2 Princess Alexandra Eye Pavilion, NHS Lothian, Chalmers St., Edinburgh EH3 9HA, UK

**Keywords:** achromatopsia, CORDs, CODs, photoreceptors, Xq28-associated disorders

## Abstract

Inherited cone disorders (ICDs) are a heterogeneous sub-group of inherited retinal disorders (IRDs), the leading cause of sight loss in children and working-age adults. ICDs result from the dysfunction of the cone photoreceptors in the macula and manifest as the loss of colour vision and reduced visual acuity. Currently, 37 genes are associated with varying forms of ICD; however, almost half of all patients receive no molecular diagnosis. This review will discuss the known ICD genes, their molecular function, and the diseases they cause, with a focus on the most common forms of ICDs, including achromatopsia, progressive cone dystrophies (CODs), and cone–rod dystrophies (CORDs). It will discuss the gene-specific therapies that have emerged in recent years in order to treat patients with some of the more common ICDs.

## 1. Introduction

**Figure 1:**
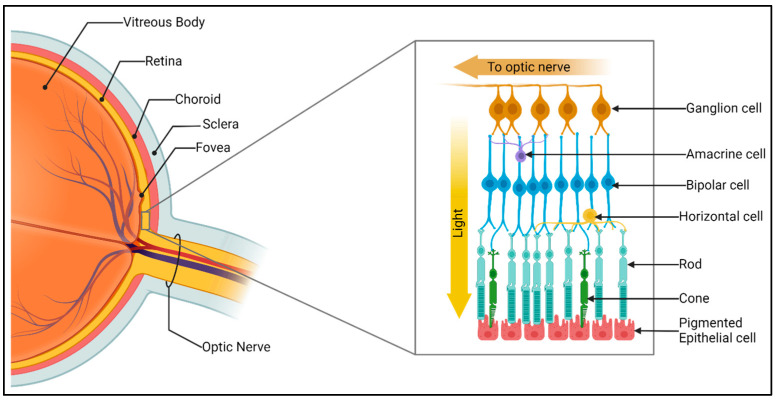
The structure of the retina. The neurosensory retina is a tri-laminar structure which sits on, and interdigitates with, the retinal pigment epithelium at the back of the eye. The optimal arrangement of the structure allows light to travel through the retina to the photoreceptors, which undergo hyperpolarization in order to initiate the visual cascade. Subsequent signalling through bipolar cells and ganglion cells (with regulation by the amacrine and horizontal interneurons) manifests with a visual impulse travelling down the optic nerve towards the brain’s occipital cortex. The information was uncovered with BioRender.com CC-BY-NC-ND.

Inherited retinal dystrophies are the leading cause of sight loss in children and working-age adults, affecting 1 in 1380 individuals [1]. Currently, over 280 genes have been identified as causing mutations that lead to the loss of the light-sensing photoreceptors in the retina [2]. The retina is a tri-laminar structure comprising 6 different cell types and it is the site of the initiation of the visual cascade (Figure 1).

**Figure 2:**
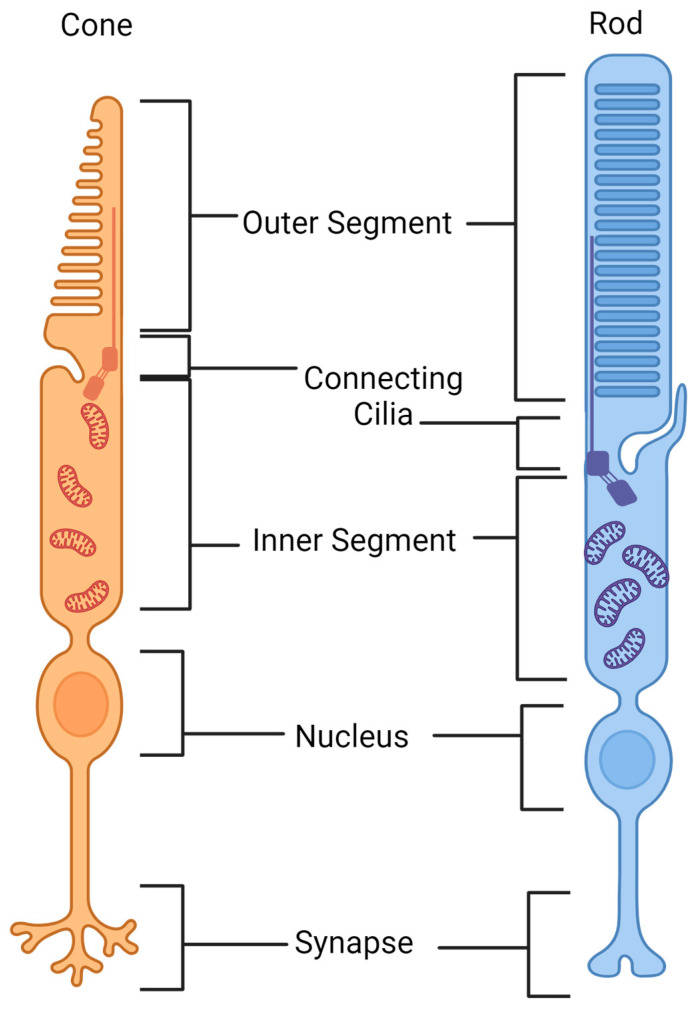
Cone and rod photoreceptors. The structures of rods and cones are similar yet distinct. Rods are much longer than cones, having more discs in their outer segment. Cones, however, are much wider. The outer segment is an extension of the photoreceptor’s specialised sensory cilium, where the ciliary membrane expands and evaginates to form disc-like formations. In rod cells, these discs fuse with and become enclosed by the overlying plasma membrane, whilst cone discs remain exposed to extracellular space. Opsins and rhodopsin (within cones and rods, respectively) are compartmentalised on the membrane of these discs, which are completely renewed every ten days via disc genesis, an actin-mediated process occurring at the connecting cilia. Discs are subsequently shed into the extracellular space at the tip of the photoreceptor for phagocytosis by the underlying RPE. The photoreceptor’s protein- and membrane-making machinery and mitochondria are located within the inner segment. This information was uncovered with BioRender.com CC-BY-NC-ND.

Photoreceptors contain a highly modified primary cilium (the connecting cilium). This has evolved to produce an expanse of folded ciliary membrane, organised into disc-like formations and termed the outer segment, in which light-sensitive opsins are concentrated [3,4,5,6] (Figure 2). These opsins undergo a conformational change upon light absorption, activating their coupled G-protein and initiating phototransduction.

There are two main types of light-sensing photoreceptor in the mammalian retina: cones and rods (Figure 2). Rod photoreceptors are responsible for vision at low light levels (scotopic vision), whilst cone photoreceptors are active at high light levels (photopic vision). Cones are capable of colour vision and are responsible for achieving high spatial visual acuity [3,4,5,6]. Humans typically have 3 types of cones, each of which detects different wavelengths of light according to the opsin they produce, and therefore afford us colour vision; *OPN1SW*, *OPN1MW* and *OPN1LW*. These encode for short-, medium- and long-wavelength opsins, respectively [3,7,8,9,10]. Cones and rods exist at varying ratios across mammalian species (1:200 in nocturnal mammals; 1:20 in primates). The primate retina is unique in its organisation, with cones concentrated in an area of the central retina known as the macula, thus offering a higher spatiotemporal resolution of vision [3]. The fovea, located at the centre of the macula, is rod-free.

Inherited cone disorders (ICDs) are a subset of IRDs that result in the dysfunction of cones and they are typically divided into two subtypes: stationary and progressive. Stationary ICDs are congenital or early-onset and give rise to purely cone dysfunction. Progressive ICDs have later onset times and usually also involve rod photoreceptors. There may be some overlap. With some developmental ICDs, such as blue-cone monochromacy (BCM), cones fail to fully develop [11,12]. ICDs are a heterogeneous group of disorders, and over 37 causal genes have now been identified. However, despite our improved understanding of ICD genetics, 43% of patients fail to receive a molecular diagnosis [13]. Patients with ICDs typically present with photophobia (possibly due to decreased cone-mediated rod inhibition) [14], decreased visual acuity, and reduced central and colour vision [13,15,16,17,18]. Diagnosis typically occurs in childhood, though it varies between disorders, with achromatopsia typically diagnosed in the first year of life, while cone–rod dystrophies (CORD) can present as late as 16 years of age [19]. With patients often affected by sight loss for their whole lives, ICDs can cause a severe burden on patients and their families.

**Figure 3:**
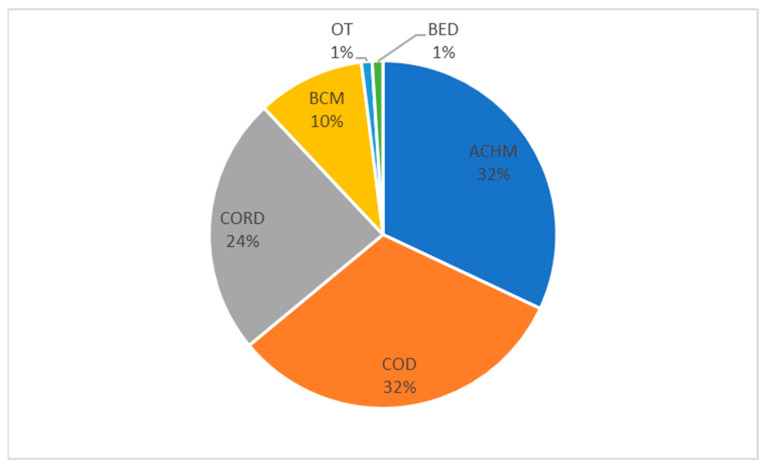
The prevalence of inherited cone disorders in the European population, (ACHM: achromatopsia, COD: cone dystrophy, CORD: cone–rod dystrophy, BCM: blue-cone monochromacy, BED: Bornholm eye disease, OT: oligocone trichromacy).

ICDs can broadly be categorised into 3 groups, achromatopsia (ACHM), cone dystrophy (COD), and cone–rod dystrophy (CORDs), each of which has a distinct mechanism of disease and clinical phenotype. Some cone diseases, such as blue-cone monochromacy and Bornholm eye disease (BED), do not fall into these groups. In Europe, ICDs have a combined prevalence of 1 in 30,000/40,000 (Figure 3), although this varies between populations [19,20,21,22,23].

## 2. Achromatopsia

ACHM is an autosomal recessive developmental condition that affects approximately 1 in 30,000 Europeans and is associated with partial or, more commonly, complete colour blindness. It is sometimes referred to as rod monochromacy, as only rods can perceive light [24]. In most cases of ACHM, whilst cones are present in the retina, they cannot respond to light [1,15,22,24,25,26,27]. Patients, therefore, present with photophobia in early infancy, reduced visual acuity (often less than 6/60), and high amplitude: low-frequency nystagmus, which can decrease in severity over time [28]. Fundoscopic examination is often unremarkable, other than a dull foveal reflex. Optical coherence tomography (OCT) can show varied structural abnormalities, including a shallow foveal dip, inner–outer-segment junction disruption and outer-nuclear-layer loss. Whilst it is not generally considered a progressive condition, longitudinal OCT studies of ACHM patients have shown age-related cone degeneration in older individuals, with four stages of ACHM proposed [29]. Adaptive optics scanning laser ophthalmoscopy (AOSLO) shows loss of the cone mosaic at the macula. Full-field scotopic electroretinography (ERG) is often unremarkable, whereas photopic testing and flicker responses are markedly attenuated [24], with multi-focal ERG (mfERG) showing reduced macular function [1,15,22,24,25,26,27].

**Figure 4:**
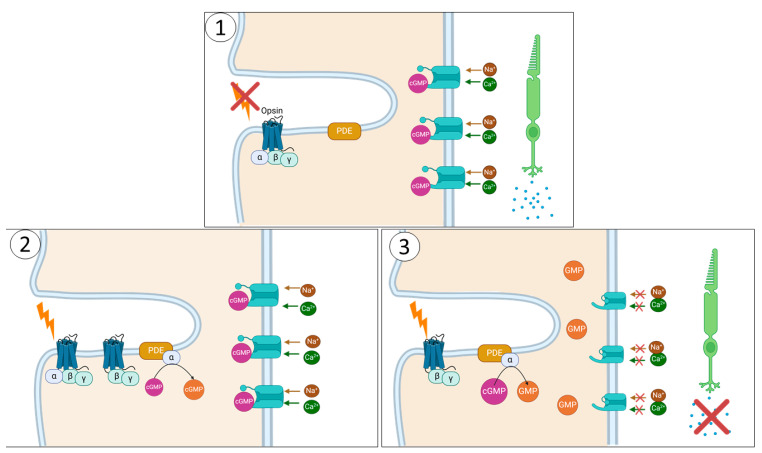
The Phototransduction cascade. The outer segment of a cone photoreceptor has disc-like evaginations in which opsins are compartmentalised. In the absence of light (1), transducin (formed of α, β, and γ subunits) remains bound to the opsin. cGMP, present in the outer segment, binds to and keeps open cGMP-gated membrane ion channels, allowing a steady influx of calcium and sodium ions into the cells called the dark current. This positive charge flowing into the cell leads to continued glutamate release from the synapse to the bipolar cells, maintaining the resting potential. When the light of a specific wavelength falls on and is detected by an opsin (2), 11-cis-retinal, which is bound to its opsin, undergoes a conformational change to being all-trans-retinal. This creates a conformational change in opsin, converting GDP into GTP, which binds the α subunit of transducin. This allows αtransducin to break away and bind the inhibitory γ subunits of phosphodiesterase (PDE), which in turn allows PDEα to hydrolyse cGMP to GMP. Reducing cGMP levels closes the ion channels (3), hyperpolarising the cell, reducing glutamate release, and allowing signalling to bipolar cells. This was determined using BioRender.com CC-BY-NC-ND.

**Figure 5:**
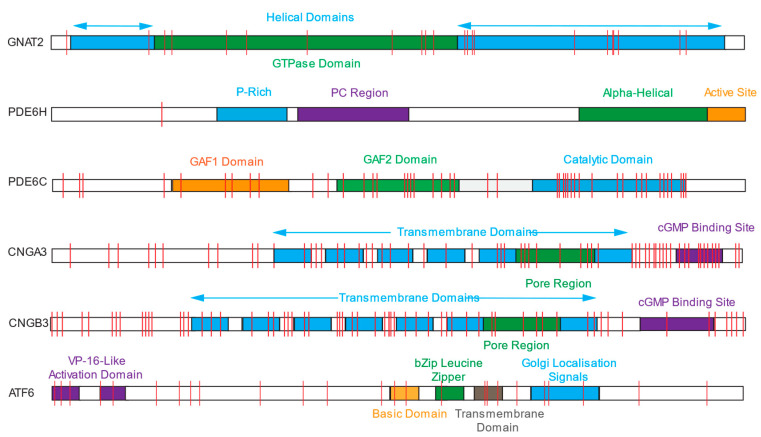
Location of pathogenic mutations on the protein domains of achromatopsia-causing genes [31].

Most ACHM is caused by variations in the genes coding for the proteins involved in the cone phototransduction cascade (Figure 4) [24], which leads to an inability to regulate the opening and closing of cyclic nucleotide-gated ion channels (CNGs). To date, six genes have been identified as having pathogenic variants which lead to ACHM, accounting for approximately 90% of cases: *CNGB3*, *CNGA3*, *GNAT2*, *PED6C*, *PDE6H*, and *ATF6* (Figure 5) [2]. Whilst 5 of these genes are involved in cone phototransduction (and their genotype-phenotype correlation is therefore expected), the transcription factor *ATF6* is a key regulator of the unfolded protein response and endoplasmic reticulum homeostasis, and the fact it plays a role in foveal development/homeostasis is surprising [30]. The prevalence of variants leading to ACHM varies geographically [24].

### 2.1. CNGB3 and CNGA

*CNGB3* encodes the β subunit of cone CNGs, whilst *CNGA3* encodes the α subunit [32]. In European populations, variations in *CNGB3* account for approximately 70% of all ACHM cases, whereas in the Middle East and China, they account for approximately 8% of cases [33]. Interestingly, the highest prevalence is on the island of Pingelap, where a typhoon in 1755 decimated the population, leaving only 20 survivors, one of which was a carrier of the p.S435F variant in *CNGB3*. This led to 10% of the population now being affected by ACHM, with a further 30% being carriers [34]. The most common variant in European populations is c.1148del, a nonsense mutation that accounts for 70% of variants of *CNGB3* [35]. *CNGA3* variants, by contrast, are more common in the Middle East and China, where they account for approximately 80% of ACHM cases. In Europe, they account for approximately 20% [33].

In cones, unlike in rods, CNGs are heteromeric tetramer channels formed of 3 α-3 subunits and one β-3 subunit [32]. If no or too little functional protein is produced, the CNGs cannot form, and calcium and sodium levels cannot be regulated in response to changes in cyclic GMP (Figure 4). This leads to an absence of phototransduction in the presence of light, and a subsequent reduction in visual acuity and colour vision.

### 2.2. PDE6C and PDE6H

*PDE6C* and *PDE6H* both encode subunits of the cone-specific cGMP phosphodiesterase, a tetramer formed of two identical α catalytic chains (encoded by *PDE6C*) [25,36] and two identical γ inhibitory subunits (encoded by *PDE6H*) [37,38]. Pathogenic *PDE6C* and *PDE6H* variants account for 2.5% and 0.1% of ACHM cases, respectively [36,37], and are thought to produce either truncated or improperly folded proteins, reducing their stability. This interrupts tetramer formation or prevents their transportation to the cell membrane [25]. In the absence of a functional PDE6, cGMP cannot be hydrolysed to GMP in response to light. CNGs remain open as a consequence, cones cannot hyperpolarize, and the phototransduction cascade therefore cannot progress further [25,38].

Patients with *PDE6C* variants have a typical ACHM phenotype, with symptoms progressing with age. Children have a relatively normal fundus appearance and minimal OCT changes. By adulthood, however, there is a reduced fovea thickness on OCT and a loss of cones on AOSLO [25,36]. Visual acuity remains stable over time [39], likely because cones are initially non-functional.

Currently, only one pathogenic variant in *PDE6H* (c.35C>G) has been identified, and it is thought to lead to the nonsense-mediated decay of shortened transcripts [37]. Three homozygous individuals in two unrelated Belgian and Dutch families have been described, with patients having incomplete ACHM with preserved short-wavelength cones [30]. Haplotype analysis suggests that this variant is a result of a mutation event of a common ancestor. The low allele frequency, with only 103 alleles being present in gnomAD [31] (91 of which are in non-Finnish European individuals), is supportive of this. Patients with this variant have been shown to have some residual colour vision and normal blue colour vision, with functioning short-wavelength cones. The mechanism behind this difference between cones has not been elucidated [37].

### 2.3. GNAT2

*GNAT2* encodes the α subunit of cone transducin. In response to light, this subunit is cleaved from the rest of the protein and binds to PDE. Variations in *GNAT2* account for approximately 1.8% of ACHM cases [15,40]. In the absence of a functional GNAT2 protein binding to PDE6, the phototransduction cascade cannot be completed. Patients with variants of *GNAT2* have varying levels of colour discrimination. Some variants result in a truncated protein being translated, with residual function, leaving patients with low-level colour vision, whilst others have none [15]. The foveal architecture of *GNAT2*-ACHM patients is relatively well maintained and they have the least disrupted photoreceptor mosaic upon treatment with AOSLO [40]. As cones are relatively well preserved, and a there is evidence that low levels of protein give rise to some colour vision, *GNAT2*-mediated disease is an ideal candidate for use in targeted gene therapy.

### 2.4. ATF6

The most recently identified ACHM gene is *ATF6*, which encodes a transcription factor that targets genes involved in the unfolded protein response during endoplasmic reticulum stress [26]. Unlike other forms, *ATF6*-ACHM patients have a near-complete absence of cones, which are stationary. Interestingly, they have increased numbers of rods [27]. Patient-derived retinal organoids do not develop cone-like structures, suggesting that ATF6 is essential for cone development. Cone development can be rescued by introducing AA147, a proteostasis regulator, to the media [41].

### 2.5. Animal Models

Though mice lack a macula, several mouse models of ACHM have been developed. *Cng3*-deficient mice display the loss of photopic ERG and cone flicker response and the loss of cone outer-segment organisation upon undergoing transmission electron microscopy [42]. Further, the loss of both *Cnga3* and *Cngb3* in mice with a cone-dominant background (loss of *Nrl*, a transcription factor required for rod development) leads to the loss of cone function upon ERG, endoplasmic reticulum stress, and early cone death [43]. *Gnat2*-deficient mice display normal cone morphology and do not undergo retinal degeneration, but exhibit the loss of cone function on photopic ERG testing [44]. The spontaneous mouse mutant, *cpfl1*, represents a homologous mouse model for *PDE6C* and develops a photopic ERG phenotype and rapid cone degeneration [45,46]. Further, a spontaneously occurring non-human primate model of *PDE6C* displays foveal thinning, progressive macular atrophy, and ERG tracing consistent with achromatopsia [47]. Not all mouse models recapitulate human disease, however. The *Atf6*^−/−^ mouse is indistinguishable from the wild-type variant until 18 months of age, when it develops the loss of both rod and cone function and pan-photoreceptor degeneration [30]. Interestingly, the *Pde6h*^−/−^ mouse displays no loss of cone structure or function, with the presence of rod-specific Pde6g detected in their cones [38]. Of note, the inverse was not observed, with *Pde6g* knock-out mice developing retinitis pigmentosa [38]. These examples highlight species-to-species variability in retinal development and neurobiology and the limitations of mice as a model organism.

## 3. *Xp28*-Associated Disorders

*Xp28* is the locus of *OPN1LW* and *OPN1MW* (there can be multiple copies of *OPN1MW*) [17]. Due to an ancestral duplication event, both genes share a locus control region (LCR) and have 98% homozygosity. Due to the high similarity between the two genes, homologous recombination between them is not uncommon [48,49]. Two different disorders have been identified that result from variants of this locus: BCM and BED.

### 3.1. Blue-Cone Monochromatism

Often grouped with ACHM due to the overlapping phenotypes of reduced visual acuity, nystagmus, photophobia, and myopia [50], BCM is the absence of medium- and long-wavelength cones due to different variants of *Xq28*. Approximately 40% of patients have different variants in the LCR region, which prevents or significantly reduces the expression of these opsins [12,18]. The remaining 60% have multiple variants and are often combinations of structural variants due to homologous recombination and inactivating mutations such as p.Cys203Arg [48,50]. All variants are X-linked-recessive, with only females being affected in cases of skewed X-inactivation [51]. Patients only have functional short-wavelength cones and thus struggle to differentiate between colours. Whilst long- and medium-wavelength cones are present, they do not respond to light. Patients typically have reduced visual acuity, ranging from 6/24 to 6/60 [18]. Over time, there is thinning of the foveola, which is long- and medium-wavelength cone-rich, suggesting that the degeneration of affected cones does occur. The patient’s phenotype, however, remains stable, likely because the cones are non-functional before degeneration [12,18].

#### Animal Models

Mice lack a long-wavelength opsin; therefore, ‘BCM’ mouse models simply reflect the loss of *Opn1mw*. Medium-wavelength cones are present in these mice, but they do not respond to light; middle-wavelength ERGs show attenuated waves with no b-wave amplitude [52]. There is also the rapid degeneration of m-cone outer segments; only 50% of m-cones are viable at 11 months [53]. BCM mouse models respond well to gene therapy approaches that deliver humanised *OPN1MW* and/or *OPN1LW* in AAV vectors. Treated mice have improved ERG and show some structural rescue. The effectiveness of the treatment is age-dependent; younger mice have a better recovery than older mice because there is less cone degeneration [53,54].

### 3.2. Bornholm Eye Disease

Bornholm eye disease is a rare ICD (<1 in 1,000,000) that was initially detected in a large family from the island of Bornholm, Denmark [13,55]. It is associated with myopia, reduced cone response in ERGs, and subnormal best-corrected visual acuity (Ranging from 20/40 to 20/80) [56]. Most patients have either protanopia (inability to detect red light due to absent OPN1LW cones) [57] or deuteranopia (inability to detect green light due to absent OPN1MW cones) [55]; this variability likely arises due to the variable function of the *Xp28* opsin genes [13]. Most patients have a mutation, resulting in the skipping of the exon 3 of either gene. There are five common SNP haplotypes: at sites 153, 171, 174, 178, and 180, we find LIAVA (Leu153; Ile171; Ala174; Val178; Ala180), LVAVA (Leu153; Val171; Ala174; Val178; Ala180), MIAVA (Met153; Ile171; Ala174; Val178; Ala180), MVAVA (Met153; Val171; Ala174; Val178; Ala180), and LIVAS (Leu153; Ile171; Val174; Ala178; Ser180). This leads to either reduced or non-functional opsin being produced, though other variants have been described [58,59].

#### Animal Model

A Bornholm eye disease mouse model has been created, with humanised *Opn1lw*/LVAVA or *Opn1lw*/LIAVA genes replacing the *Opn1mw* gene. Further, *Opn1sw* was also knocked out in the strain, as some murine cones co-express medium- and short-wavelength opsins [60,61,62]. The *Opn1lw*/LVAVA model developed a mild cone dystrophy, with L/M cone ERG amplitudes reduced by 50% at all measured time points (2–16 months). Furthermore, cone outer-segment lengths were shorter (16.1% at 3 months and 23.2% at 6 months) compared to the control [61]. The *Opn1lw*/LIAVA model, conversely, did not have significantly attenuated L/M cone ERG amplitudes and no change was observed in outer-segment length. Of note, more cone opsin is present in *Opn1lw*/LIAVA mice than in *Opn1lw*/LVAVA mice [61]. The slow cone dystrophy of LVAVA suggests it may be amenable to use in gene therapies [60].

## 4. Cone and Cone–Rod Dystrophies

**Figure 6:**
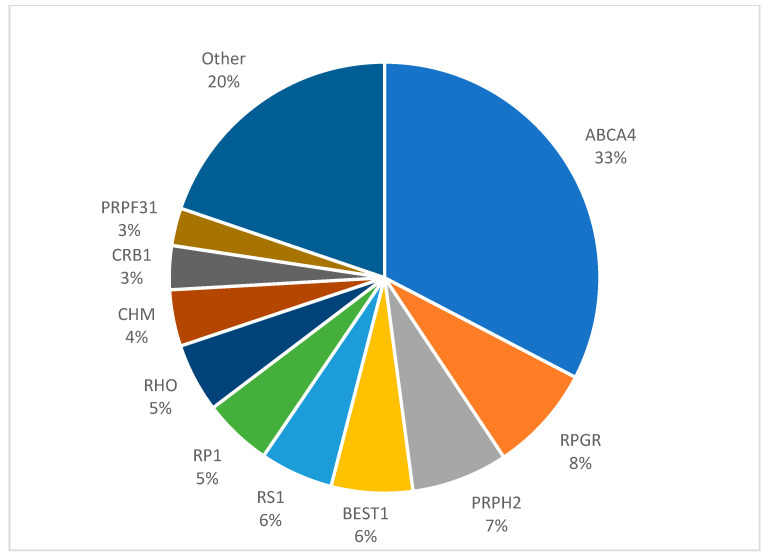
Gene prevalence in cone and cone–rod dystrophies in European populations.

CODs and CORDs are heterogeneous groups of disorders that each occur in approximately 1 in 30,000 Europeans. They are characterised by the degeneration of cones in isolation or of the degeneration of cones followed by the loss of rods, respectively. During adolescence, patients present with reduced central vision, abnormal colour vision, and photophobia. Nystagmus is not commonly observed [20,63]. Nyctalopia can also develop in CORD patients and, eventually, in COD patients at a late stage. The age of onset varies depending on the affected gene and the causal mutation, with an average age of diagnosis of 12 years for CORD and 16 years for COD patients, respectively [19]. Fundoscopic examination can show a bull’s eye maculopathy, whilst OCT imaging shows the progressive loss of outer segments of the macula and, in the case of CORD and later-stage COD, throughout the retina. AOSLO shows changes in the mosaic at the macula and the emergence of ‘dark cones’; it is proposed that they are hyporeflective in nature (relative to surrounding rods) due to the loss of their outer segments [64]. Scotopic ERGs can show attenuated red flash b waves_x_, whilst scotopic blue and white flash b waves have a delayed but normal peak. Phototopic testing and flicker responses are markedly attenuated or absent [64]. As COD and CORDs progress, scotopic blue and white ERG responses are reduced over time. This occurs earlier in CORDs. Variants of 26 genes [2] have been found to cause CODs and CORDs (Figure 6), with certain types being more likely to be identified. Most recently, the ubiquitin-associated protein 1-like gene (*UBAP1L),* a gene of unknown function, has been identified as a novel cause of CODs and CORDs in a diverse patient sample [65,66]. For this review, however, we focus on 3 common genes: *ABCA4, PRPH2*, and *RPGR.*

### 4.1. ABCA4

Approximately 25% of CODs and CORDs worldwide result from pathogenic variants of the ATP-binding cassette A4 (*ABCA4*) [13]. *ABCA4* encodes a protein which prevents the build-up of N-retinylidene phosphatidylethanolamine (NrPE) in the photoreceptor. NrPE is formed when phosphatidylethanolamine (PE) reacts with all-trans-retinal (ATR), a by-product of the phototransduction cascade. ABCA4 internalises NrPE from the photoreceptor disc lumen to the cytoplasm, allowing it to be hydrolysed back to ATR and PE. In its absence, NrPE remains on the lumen side of the disc membrane and condenses with ATR to form the hydrophobic, cytotoxic fluorophore known as A2E. Following disc shedding and phagocytosis by the RPE, A2E accumulates in lipofuscin granules in the RPE, leading to cell stress and death [67]. Over 1000 disease-causing variants in *ABCA4* have been identified; these lead to various recessive conditions, the most common being Stargardt disease [68]. Stargardt disease is characterised by RPE death at the macula, followed by the degeneration of the overlying photoreceptors [67]. In null mutations, cone death tends to occur first [35,68] and patients present the progressive loss of central vision in childhood. Blue-light autofluorescent imaging shows areas of RPE lipofuscin accumulation, as evidenced by hyperautofluorescence, and surrounding areas of RPE death, as evidenced by hypoautofluorescence. OCT imaging shows RPE death at the macula, with overlying photoreceptor degeneration; AOSLO imaging also shows the wider spacing of cones in *ABCA4* patients, with the widest spacing occurring in the perifovea [69]. The b-wave of the photopic ERG is markedly attenuated, whilst the b-wave of the scotopic ERG is initially normal, decreasing as the disease progresses [70,71]. Cone involvement is often followed by rod cell death due to progressive A2E accumulation. Patients can also develop an *ABCA4*-associated CORD, which presents as a typical Stargardt’s phenotype with an additional paracentral scotoma and constricted peripheral fields. The median onset is lower than in other CORDs at the age of 9 years.

### 4.2. PRPH2

*PRPH2* variants account for 5.2% of IRDs in the United Kingdom and account for 20% of autosomally dominant ICDs [13,72]. *PRPH2* encodes Peripherin-2, a cell surface glycoprotein that is a member of the tetraspanin family. It is essential for the morphogenesis of outer-segment discs in photoreceptors and maintenance of disc rim curvature and is thought to play a role in their stabilisation and compaction [72,73], preventing the release of ectosomes from the photoreceptor CC [74]. *PRPH2*-associated CORDs are characterised by a speckled macula appearance on fundus images, with the development of a bull’s eye maculopathy and eventual macular atrophy. Some patients have wider-spaced cones on AOSLO images [75]. However, there is considerable phenotypic variability, even between individuals from the same family [76]. Variants in *PRPH2* can lead to retinitis pigmentosa, as well as to CORDs [72]. The nature of the IRD is allele-dependent, with dominant negative and gain-of-function variants leading to a cone-dominant phenotype, whilst haploinsufficiency variants lead to rod-dominant phenotypes [77]. The exact mechanism behind this difference has yet to be elucidated.

### 4.3. RPGR

Variants of *RPGR* account for approximately 70% of X-linked IRDs. *RPGR* is an alternatively spliced gene. The major isoform, RPGR1-19, is encoded by exons 1–19 and is constitutively expressed [78,79,80]. The retina-specific isoform, RPGRORF15, contains exons 1–15 before splicing them into intron 15, leading to a 1152 amino acid protein [80] containing a repetitive, disordered Glu-Gly rich domain of unknown function, followed by a basic domain [4,78,81]. RPGR appears to play a role in outer-segment maintenance [82]. Nonsense or truncating mutations predominantly cause X-linked retinitis pigmentosa (XLRP) [78,81], but rod–cone or cone–rod dystrophies can develop [83,84]. The location of the variants seemingly affects the phenotype of *RPGR* mutations, with disease-causing mutations within exons 1–14 predominantly causing retinitis pigmentosa, truncating mutations within ORF15 leading to CORD, and mutations in the C-terminal basic domain causing a COD phenotype [85]. It is thought that the glutamylation of RPGRORF15 occurs through the binding of TTLL5 to the basic domain and thus truncated variants have impaired glutamylation, affecting cones to a greater degree than rods [85,86]. Those with a COD or CORD initially present with decreased visual acuity or photophobia. Fundal examination shows that the hypoautofluorescence of the macula and OCTs shows the thinning of the retina at the macula. ERGs show a decreased (by 1.3–20% of normal lower limit) photopic, response with some patients having near-complete loss of photopic responses; there is also some reduction in the scotopic response. Furthermore, the 30 Hz flicker gives no/very little measurable response [16]. The average age of onset is 25 years, reaching legal blindness by 42 years [83,84].

### 4.4. Animal Model

Several mouse models of COD and CORD have also been developed. *Rpgr* knock-out mice, with an 8 base pair deletion in exon 3, show a decrease in the photopic, cone-associated b wave at 6 months (similar to that seen in patients), with loss of the outer nuclear layer occurring later [4]. Fundal examination also shows punctate lesions and increased autofluorescence. Of note, some mutations cause different phenotypes depending on background strain used. An in-frame deletion of exon 4 on a Bl6 background gave a rod-dominant phenotype characterised by a decreased scotopic a-wave from 3 months and a marginally decreased photopic b-wave from 9 months, whilst the scotopic b wave was unaffected. Conversely, the same mutation on a BALB/c background resulted in a cone-dominated phenotype, characterised by a reduced photopic b wave at 1 month [87]. Morphometric analysis showed decreased thickness of the outer and inner segments in the BL/6 mice, whilst there was no difference in the BALB/c mice [87]. This highlights the impact that background strain can have on IRD models.

*Prph2* mice with an R172W mutation exhibit a dominant negative CORD phenotype, characterised by the thinning of the outer nuclear layer and decreased photopic b-wave and (to a lesser degree) scotopic a-wave from p30. When a functional copy of *Prph2* was introduced, rods, but not cones, were rescued [88]. A humanised, *K153Δ-Prph2* knock-in mouse, replicating a pathogenic human variant, also showed a CORD phenotype, with significantly reduced scotopic a and b waves from p30 and the absence of outer segments. Heterozygous K153Δ mice showed shorter outer segments and reduced rhodopsin, constituting approximately 59% of WT levels. This phenotype was milder than that seen in heterozygous *Prph2* knock-out mice, which exhibit a whirling of the OS. Complete *Prph2* knock-out mice demonstrate a severe phenotype, marked by the reduced presence of outer segments and severe whirling. ERG analysis shows the significant attenuation of the scotopic a and b waves as well as photopic b-wave amplitudes in heterozygous *Prph2* knock-out mice at both p30 and p180 [89]. Notably, the overexpression of wild-type *Prph2* in these mice rescues the photopic b-wave amplitude and increases the amplitude of scotopic a and b waves at p30; however, at p180 there is no significant difference in ERG responses. Furthermore, in these mice, outer-segment morphology at p30 is improved. This suggests that the overexpression of PRPH2 may delay the CORD phenotype; however, it does not rescue it completely due to the dominant negative effect of the K153Δ genotype [89].

*Abca4*^−/−^ mice exhibit RPE atrophy but normal photoreceptors with no outer-segment disorganisation [90], possibly as the rate of all-trans-retinal clearance in these mice is comparable with wild-type mice [91]. The attenuation of all-trans-retinal clearance is observed in *Rdh8*^−/−^*Abca4*^−/−^ mice, which leads to an accumulation of A2E and photoreceptor outer-segment degeneration. Cone degeneration is observed in these mice, with less than five cones/100 µm (~25 cones/100 µm in wild-type variant). ERGs at 3 months showed significantly attenuated scotopic a and b wave amplitudes. In these mice, there was also degeneration of the RPE as in *Abca4*^−/−^ mice [91].

## 5. Gene Therapies

The eye has been a model organ in the development of gene therapy technologies due to it being easily accessible, readily visualized, and relatively immune-privileged. As a result, FDA- and EMA-approved gene therapy products exist for a rare form of IRD, and so the technology offers hope in terms of developing treatments for ICDs. Presently, sub-retinal delivery is the accepted delivery route, though, in the longer term, the development of effective products that can be delivered via an intravitreal route is desirable, given the lower risk profile and commonality of the approach [92,93]). AAV vectors are commonly used for gene delivery as they allow for long-term gene expression from one injection and have low immunogenicity. AAV serotypes 2/5, 2/7, 2/8 and 2/9 efficiently transduce photoreceptors [92]. Lentiviral vectors offer a larger carrying capacity than AAV vectors (−7 kb vs. 4.7 kb), but concerns remain about the tumourigenic potential of the technology from the random integration into the host genome [92,94,95].

For patients with ACHM, there are currently several gene therapies in trial. These target *CNGB3* and *CNGA3*, utilising AAV vectors to deliver human copies of the genes (Table 1). Some phase I/II results have been published. Reichel et al., 2022 administered AAV.*CNGA3* gene therapy to one eye of 9 adults with ACHM, demonstrating a good safety profile. However, there was no significant improvement in most secondary endpoints. The group postulated that its potential might be heightened in children, given their greater brain plasticity [96]. In 2022, Farahbakhsh et al. trialled AAV.*CNGA3* and AAV.*CNAGB3* gene therapies that were delivered to children aged 10–15. The treatments demonstrated safety, with two out of four patients displaying enhanced cone function [97]. However, cone function was lower than normal levels. Michaelides et al., 2023 trialled an AAV8-hCARp.*hCNGB3* gene therapy (NCT03758404) in 11 children and 23 adults. The treatment was deemed safe and there was an improvement in some individuals, with 21/23 reporting improved vision-related quality of life [98]. Current gene therapy trials for ACHM exhibit variable success and safety profiles across age groups, though achieving optimal functional levels remains a challenge.

For the cone and cone–rod dystrophies, gene therapy trials for *ABCA4*- and *RPGR*-mediated disease, focusing on Stargardt and Retinitis Pigmentosa, respectively, are currently underway (Table 1) [99]. *ABCA4* is a large gene (6.8 kb) [94] and thus a single AAV vector cannot be used to deliver the whole gene. Lentiviral vectors have been developed and shown to be safe. However, the study into them was terminated early as no visual improvement was observed and 27% of patients developed worsening RPE atrophy [94,95]. Lipid-nanoparticle-encasing *ABCA4* gene therapy has also been developed and trialled in mice, where it has been shown to be safe. The delivery of 100 ng/eye resulted in a 125-fold increase in the expression of ABCA4 at 4 months, remaining significant at 1 year. The study also showed reduced A2E expression from 4 months to 1 year; with 3 treatments over 1 year, there was a 70% reduction in A2E accumulation compared to the control. Repeat injections also prolonged the expression of ABCA4 compared to just one [100]. To circumvent the AAV vector capacity limit, multiple AAV vectors can be used to load transgene cassettes which split the gene using split intein-mediated trans-splicing technology. Using this method, post-translational intein excision and concomitant ligation allow for the reassembly of the parts of the gene into a full-length protein in the target cell. This method allows for 70% expression levels of wild-type Abca4 protein in *Abca4*^−/−^ murine retina and, at one-year post-treatment, leads to significant reduction in A2E levels and recovery of ERG responses, suggesting this could be a viable therapeutic approach for Stargardts patients [101].

**Table 1:** Clinical Trials of Gene Therapies [100].

NCT Number	Study Title	Interventions	Phases	Start Date	Completion Date	Study Status
*CNGA3*						
NCT02610582	Safety and Efficacy of rAAV.h*CNGA3* Gene Therapy in Patients With *CNGA3*-linked Achromatopsia	rAAV.h*CNGA3*—gene therapy	PHASE1|PHASE2	November 2015	June 2027	Active
NCT03278873	Long-Term Follow-Up Gene Therapy Study for Achromatopsia *CNGB3* and *CNGA3*	AAV—*CNGA3*—gene therapy	PHASE1|PHASE2	29 June 2017	4 April 2024	Completed—no results published
NCT03758404	Gene Therapy for Achromatopsia (*CNGA3*)	AAV—*CNGA3*—gene therapy	PHASE1|PHASE2	12 August 2019	10 June 2021	Completed—results published [98]
NCT02935517	Safety and Efficacy Trial of AAV Gene Therapy in Patients With *CNGA3* Achromatopsia (A Clarity Clinical Trial)	AGTC-402—gene therapy	PHASE1|PHASE2	3 August 2017	August 2026	Active
*CNGB3*						
NCT02599922	Safety and Efficacy Trial of AAV Gene Therapy in Patients With *CNGB3* Achromatopsia (A Clarity Clinical Trial)	rAAV2tYF-PR1.7-h*CNGB3*—gene therapy	PHASE1|PHASE2	11 April 2016	July 2026	Active
NCT03278873	Long-Term Follow-Up Gene Therapy Study for Achromatopsia *CNGB3* and *CNGA3*	AAV—*CNGB3*—gene therapy	PHASE1|PHASE2	29 June 2017	4 April 2024	Completed—no results published
NCT03001310	Gene Therapy for Achromatopsia (*CNGB3*)	AAV—*CNGB3*—gene therapy	PHASE1|PHASE2	16 January 2017	25 October 2019	Completed—results published [96]
*RPGR*						
NCT04794101	Follow-up Gene Therapy Trial for the Treatment of X-linked Retinitis Pigmentosa Associated With Variants in the *RPGR* Gene	AAV5-hRKp.*RPGR*—gene therapy	PHASE3	4 December 2020	19 December 2029	Active
NCT03316560	Safety and Efficacy of rAAV2tYF-GRK1-*RPGR* in Subjects With X-linked Retinitis Pigmentosa Caused by *RPGR* Mutations	rAAV2tYF-GRK1-*RPGR*—gene therapy	PHASE1|PHASE2	16 April 2018	March 2025	Active
NCT05874310	Gene Therapy for Subjects With *RPGR* Mutation-associated X-linked Retinitis Pigmentosa	FT-002—gene therapy	EARLY_PHASE1	1 February 2023	1 November 2027	Recruiting
NCT06275620	A Study Comparing Two Doses of AGTC-501 in Male Participants With X-linked Retinitis Pigmentosa Caused by *RPGR* Mutations (DAWN)	AGTC-501—gene therapy	PHASE2	14 November 2023	August 2029	Enrolling
NCT04671433	Gene Therapy Trial for the Treatment of X-linked Retinitis Pigmentosa Associated with Variants in the *RPGR* Gene	AAV5-hRKp.*RPGR*—gene therapy	PHASE3	4 December 2020	20 September 2024	Active
NCT04850118	A Clinical Trial Evaluating the Safety and Efficacy of a Single Subretinal Injection of AGTC-501 in Participants With XLRP	rAAV2tYF-GRK1-h*RPGR*co G	PHASE2|PHASE3	14 March 2024	October 2029	Recruiting
NCT04517149	4D-125 in Patients With X-Linked Retinitis Pigmentosa (XLRP)	4D-125—gene therapy	PHASE1|PHASE2	9 June 2020	May 2029	Active
NCT03584165	Long-term Safety and Efficacy Follow-up of BIIB111 for the Treatment of Choroideremia and BIIB112 for the Treatment of X-Linked Retinitis Pigmentosa	BIIB111/BIIB112—gene therapy	PHASE3	4 June 2018	4 June 2026	Enrolling
NCT03252847	Gene Therapy for X-linked Retinitis Pigmentosa (XLRP)—Retinitis Pigmentosa GTPase Regulator (*RPGR*)	AAV2/5-*RPGR*—gene therapy	PHASE1|PHASE2	14 July 2017	18 November 2021	Completed—no results published
NCT05926583	A Study of AAV5-hRKp.*RPGR* for the Treatment of Japanese Participants With X-linked Retinitis Pigmentosa	AAV5-hRKp.*RPGR*—gene therapy	PHASE3	12 September 2023	9 October 2029	Recruiting
NCT03116113	A Clinical Trial of Retinal Gene Therapy for X-linked Retinitis Pigmentosa Using BIIB112	BIIB112—gene therapy	PHASE1|PHASE2	8 March 2017	18 November 2020	Completed—Results Published [85]
NCT06333249	A Study Comparing Two Doses of AGTC-501 in Male Subjects With X-linked Retinitis Pigmentosa Caused by *RPGR* Mutations (SKYLINE)	rAAV2tYF-GRK1-*RPGR*—gene therapy	PHASE2	13 April 2021	February 2027	Active
*ABCA4*						
NCT01367444	Phase I/IIA Study of SAR422459 in Participants With Stargardt’s Macular Degeneration	SAR422459—EIAV-*ABCA4* gene therapy	PHASE1|PHASE2	8 June 2011	16 August 2019	Terminated due to adverse effects [94,95]
NCT06300476	Safety and Efficacy of a Single Subretinal Injection of JWK006 Gene Therapy in Subjects With Stargardt Disease (*STGD1*)	JWK006—AAV-*ABCA4* gene therapy	PHASE1|PHASE2	20 November 2023	30 December 2029	Active

*RPGR*, a smaller gene than *ABCA4*, can be packaged into a single AAV vector and several gene therapy products are undergoing trials for RPGR-mediated disease, though it must be stressed they currently focus on *RPGR*-mediated retinitis pigmentosa rather than cone dystrophies [99]. A codon-optimised *RPGR* has been trialled in an AAV8 vector, with the phase I/II trial reporting an increased retinal sensitivity in the treated eye, as evidenced by microperimetry, increasing over time up to 6 months [85]. The phase II/III study did not meet its primary endpoint. It remains to be seen whether such an approach can be used to effectively treat *RPGR*-mediated cone dystrophies.

### Clinical Heterogeneity

The marked clinical heterogeneity observed in *RPGR*-mediated disease is not unique to this gene. Mutations in many ICD causal genes can lead to a pure rod dystrophy without cone involvement. Further, mutations in several ICD genes can result in syndromic disease. The photoreceptor outer segment is a highly modified primary cilium and thus variants in genes involved in the formation or maintenance of primary cilia can result in syndromic ICDs. Different variants in *CC2D2A* have been shown to cause a range of phenotypes, including Joubert Syndrome and Meckel Syndrome, along with retinitis pigmentosa and CORDs [102,103]. Similarly, mutations in the Bardet Biedel gene family can alternatively cause Bardet Biedel syndrome or an isolated CORD [104,105].

## 6. Conclusions

Inherited cone disorders affect approximately 1 in 10,000 individuals and are the result of dysfunction in our cone photoreceptors, responsible for the perception of colour vision. Efforts have been made to distinguish between subsets clinically, though there remains significant phenotypic overlap. Although 37 causal genes have been identified, over half of patients have no confirmed genetic diagnosis. Given the emergence of gene-based therapeutic approaches to target specific ICDs, achieving an improved understanding of the molecular mechanisms underpinning these diseases and an improved molecular diagnosis rate is paramount.

## 7. Limitations of the Review

Whilst we attempted to cover all causal genes for ICDs in this review, it is difficult to be entirely comprehensive and there is a risk that the literature search was not exhaustive. Some studies may have been missed. Further, emerging clinical studies may have been overlooked. Novel genes and studies published at the time of submission are particularly at risk of this.
